# Clinical Prediction Score for Ruptured Appendicitis in ED

**DOI:** 10.1155/2021/6947952

**Published:** 2021-03-12

**Authors:** Thidathit Prachanukool, Chaiyaporn Yuksen, Welawat Tienpratarn, Sorravit Savatmongkorngul, Panvilai Tangkulpanich, Chetsadakon Jenpanitpong, Yuranun Phootothum, Malivan Phontabtim, Promphet Nuanprom

**Affiliations:** Department of Emergency Medicine, Faculty of Medicine, Ramathibodi Hospital, Mahidol University, Bangkok 10400, Thailand

## Abstract

**Background:**

Ruptured appendicitis has a high morbidity and mortality and requires immediate surgery. The Alvarado Score is used as a tool to predict the risk of acute appendicitis, but there is no such score for predicting rupture. This study aimed to develop the prediction score to determine the likelihood of ruptured appendicitis in an Asian population.

**Methods:**

This study was a diagnostic, retrospective cross-sectional study in the Emergency Medicine Department of Ramathibodi Hospital between March 2016 and March 2018. The inclusion criteria were age >15 years and an available pathology report after appendectomy. Clinical factors included gender, age>60 years, right lower quadrant pain, migratory pain, nausea and/or vomiting, diarrhea, anorexia, fever>37.3°C, rebound tenderness, guarding, white blood cell count, polymorphonuclear white blood cells (PMN) > 75%, and pain duration before presentation. The predictive model and prediction score for ruptured appendicitis were developed by multivariable logistic regression analysis.

**Result:**

During the study period, 480 patients met the inclusion criteria; of these, 77 (16%) had ruptured appendicitis. Five independent factors were predictive of rupture, age>60 years, fever>37.3°C, guarding, PMN>75%, and duration of pain>24 hours to presentation. A score >6 increased the likelihood ratio of ruptured appendicitis by 3.88 times.

**Conclusion:**

Using the Ramathibodi Welawat Ruptured Appendicitis Score (RAMA WeRA Score) developed in this study, a score of >6 was associated with ruptured appendicitis.

## 1. Introduction

Acute appendicitis is a surgical emergency that must be treated by urgent surgery within 24–48 hours of the onset of acute abdominal pain [1, 2]. The presentation can occur in patients of any age or gender, with risks of approximately 8.6% in males, 6.7% in females, [3, 4] 90% in children, and 10% in the elderly [3, 5]. Rupture is a serious complication of acute appendicitis and typically occurs in 17%–20% of cases, increasing to 45% in children younger than 5 years and to 51% in patients older than 65 years. A ruptured appendicitis was associated with high morbidity and mortality, especially in the elderly [6].

At present, the Alvarado Score is being used as a prediction score to determine the likelihood of acute appendicitis based on symptoms, signs, and laboratory results. The total possible score is 10, with 1–4 points indicating a 30% risk (allowing discharge), 5–6 indicating a 66% risk (necessitating observation), and 7–10 indicating a 93% risk (necessitating admission for surgery) [2, 7]. Ultrasound and computed tomography (CT) are considered to aid diagnosis. Ultrasound only has a sensitivity of 44%–98% and a specificity of 47%–95% [8–10]. Although CT offers superior diagnosis and vision, ultrasound is associated with fewer risks and is noninvasive, requiring no injection of contrast media [11].

A ruptured appendicitis was associated with older age, sex (male), duration from the abdominal pain onset to presentation, fever (>38°C), diarrhea, leukocytosis, and the left shift [4–6, 12–18]. The objective of this research was to evaluate clinical factors associated with ruptured appendicitis and develop the prediction score to determine the likelihood of ruptured appendicitis.

## 2. Method

This study was a diagnostic, retrospective cross-sectional study in the Emergency Medicine Department of Ramathibodi Hospital, a university-affiliated super tertiary care hospital in Bangkok, Thailand. We included patients with the following ICD-10 final diagnoses: K35.9 acute appendicitis (unspecified) and K35.0 acute appendicitis (with generalized peritonitis). The study period was 2 years starting from March 2016 to March 2018. The eligible criteria were aged >15 years, being diagnosed with acute appendicitis in emergency department (ED), and to have pathological results available following appendectomy. We excluded those with no pathology report.

The study variables were recorded for all eligible patients, including the baseline characteristic factor and potential clinical factors for ruptured appendicitis. Clinical factors included gender, age>60 years [5], right lower quadrant pain, migratory pain, nausea and/or vomiting, diarrhea, anorexia, fever>37.3°C, rebound tenderness, guarding, white blood cell count, polymorphonuclear white blood cells (PMN) > 75%, and pain duration before presentation.

The outcome was a positive pathological result for ruptured or perforated appendicitis, as reported by a pathologist. Patients were then categorized into either a ruptured appendicitis group or nonruptured appendicitis group including inflammation, suppurative, and gangrenous. Finally, we developed a risk score, which we entitled the Ramathibodi Welawat Ruptured Appendicitis Score (RAMA WeRA Score).

### 2.1. Statistical Analysis

Data were analyzed by using STATA version 14.0. We followed the methods of Yuksen et al. [19]. All study variables were compared between the ruptured and nonruptured groups by descriptive statistics. The potential predictors were compared to identify differences (*p* value) in clinical characteristics using *t*-test and exact probability test. The predictive factors were individually calculated via a univariate logistic regression analysis and were presented as an area under the receiver operating characteristic (AUROC) curve and 95% confidence interval (95% CI). The clinical predictors that had a high discriminative performance (AUROC curve), *p* value, and clinical relevance were divided into two categories by calculating odds ratio (OR) via a multivariate logistic regression analysis.

The regression coefficients for each category of the clinical predictor were divided by the smallest coefficients of the model and were rounded off to the nearest 1 to transform into an item risk score. The coefficients were transformed to item risk scores and were summed up to a single score. On the basis of the scores, the patients were classified under the low, moderate, and high probability categories.

### 2.2. Ethical Considerations

This study was approved by the Faculty of Medicine, Committee on Human Rights Related to Research Involving Human Subjects of Mahidol University's Ramathibodi Hospital. The need for informed consent was waived by the ethics committee due to retrospective design.

## 3. Results

During the study period, there were 480 patients who met the study criteria, of whom 77 (16%) had pathologically confirmed ruptured appendicitis. The nonruptured appendicitis group was younger (41.05 ± 17.75 vs. 51.98 ± 19.55 years; *p* < 0.001). Five factors had high discriminative performance (AUROC curve) and were significantly associated with the pathological results (Table 1): age>60 years, fever>37.3°C, guarding, PMN>75%, and duration of pain>24 hours to presentation. Multivariate analysis confirmed that each of these was predictive of positive pathological results (ruptured appendicitis), age>60 years with an adjusted OR of 2.20 (95% CI: 1.20, 4.02), fever>37.3°C with an adjusted OR of 2.28 (95% CI: 1.26, 4.12), guarding with an adjusted OR of 3.78 (95% CI: 2.16, 6.62), PMN>75% with an adjusted OR of 2.31 (95% CI: 1.33, 8.52), and duration of pain to ER > 24 hours with an adjusted OR of 6.20 (95% CI: 2.28, 19.16). The scores ranged from 0 to 3 (Table 2).

The score-predicted risk increased in close association with the observed risk. Finally, the risk scores were categorized into three groups: score <2 (low risk), scores 2–6 (moderate risk), and score >6 (high risk). The positive likelihood ratio of ruptured appendicitis in the high-risk group was 3.88. The risk score was named as RAMA WeRA Risk Score.

The accuracy of RAMA WeRA Risk Score for predicting ruptured appendicitis was investigated (Table 3). In low-risk patients, specificity was 88.8%, NPV was 82.3%, and LR+ was 0. In moderate-risk patients, sensitivity was 40.3%, specificity was 26.6%, PPV was 9.5%, NPV was 70%, and LR+ was 0.55. For those of high risk, sensitivity was 59.7%, specificity was 84.6%, PPV was 38.9%, NPV was 91.7%, and LR+ was 3.88.

In the high-risk group, 31/372 patients had false negative for ruptured appendicitis, which resulted in delays in management. 62/108 patients had false positive for ruptured appendicitis, which resulted in unnecessary stress to patients.

## 4. Discussion

Several clinical prediction criteria have been reported in Asian countries for determining the risk of ruptured appendicitis [5, 6]. We found that these clinical predictors in cases of suspected acute appendicitis were similar in a Thai population in terms of age >60 years, fever, guarding, PMN, duration to ER, anorexia, gender, and male (Table 4). Our score also presented the risk as a more user-friendly probability score that can be used without radiological data. Ruptured appendicitis was found in cases where patients with suspected acute appendicitis had moderate or high scores (>6) (Table 3). If the RAMA WeRA Risk Score exceeds 6 points, we should be aware that a ruptured appendicitis is possible and immediately refer for appropriate management. This may be ultrasound, CT, or surgical consultation and admission depending on local facilities [1]. Such a score should also provide sufficient justification to start empirical antibiotics (ceftriaxone plus metronidazole) in patients with sepsis/peritonitis or who are hemodynamically unstable [20, 21].

We identified three predictors for rupture in patients with acute appendicitis that were similar to those found in previous reports [5, 6]. These were age >60 years, fever, and time to presentation, as shown in Table 4. However, unlike other reports [5, 6], gender (male) and anorexia were not significant predictors of ruptured appendicitis. The nonsignificance of anorexia may be explained by this information not being recorded due to the retrospective data collection. Gender (male) was also not a significant predictor of ruptured appendicitis.

Fever >37.3°C and PMN >75% were predictors of rupture in this study and are used to predict acute appendicitis in the Alvarado Score. Thus, only the remaining three predictors (age, guarding, and time to presentation) additionally predicted ruptured appendicitis when acute appendicitis was already suspected (Alvarado Score >4). In a previous report [5], the Alvarado Score was analyzed as a predictor for ruptured appendicitis but was not significant and could not be used for those younger than 60 years. By contrast, the RAMA WeRA Risk Score is able to predict ruptured appendicitis with an accuracy of 81%.

There are limitations to this study. First, this study was retrospective data collection and conducted in a single center. Another limitation was that we included all types of appendicitis as nonruptured appendicitis, including inflammation, suppurative, and gangrenous. We did not include the operative findings in the result. However, the proposed RAMA WeRA Risk Score was based on clinical and basic laboratory investigations and can enable more accurate preoperative classification of patients by rupture status. We now need to validate our results externally to establish the true value of our risk score for management decisions.

In conclusion, the RAMA WeRA Risk Score provides a screening tool for predicting rupture in patients with suspected acute appendicitis. A clinical predictive score of >6 appears to be associated with rupture of the appendix in cases of acute appendicitis.

## Figures and Tables

**Table 1:** Clinical characteristics of participants categorized by pathology results as ruptured or nonruptured appendicitis.

Characteristics	Ruptured (+) (*n* = 77)		Nonruptured (−) (*n* = 403)		*p* value	AUROC (95% CI)
	*n*	%	*n*	%		
Gender, male	34	44.16	163	40.45	0.613	0.52 (0.46,0.58)
Age>60 years	30	38.96	71	17.62	<0.001	0.61 (0.55,0.66)
RLQ pain	77	100	402	99.75	1.000	0.501 (0.498,0.503)
Migratory pain	42	54.55	240	59.55	0.449	0.48 (0.41,0.53)
Nausea and vomiting	43	55.84	242	60.05	0.528	0.48 (0.41,0.54)
Diarrhea	15	19.48	90	22.33	0.653	0.59 (0.44,0.53)
Anorexia	37	48.05	162	40.20	0.209	0.54 (0.49,0.66)
Fever>37.3°C	55	71.43	195	48.39	<0.001	0.62 (0.59,0.67)
Rebound tenderness	56	72.73	280	69.48	0.684	0.52 (0.46,0.57)
Guarding	48	62.34	103	25.56	<0.001	0.68 (0.63,0.74)

WBC						
≤10000	14	18.18	55	13.65	0.531	0.48 (0.42,0.55)
10000–15000	32	41.56	184	45.66		
>15000	31	40.26	164	40.69	0.014	0.56 (0.52,0.60)
PMN > 75%	69	89.61	311	77.17		

Duration of pain to ER						
<12 hours	4	5.19	98	24.32	<0.001	0.65 (0.62,0.70)
12–23 hours	2	2.60	30	15.38		
>24 hours	71	92.21	243	60.30		

*Note*. Nonruptured (−) refers to patients without rupture/perforation of appendicitis (inflammation, suppurative, and gangrenous). AUROC, area under the receiver operating characteristic curve; RLQ pain, right lower quadrant pain; WBC, white blood cell count; PMN, polymorphonuclear white blood cell.

**Table 2:** Predictors of ruptured appendicitis and the assigned item score in cases of suspected acute appendicitis.

Predictors	Category	aOR	95% CI	*p* value	Coefficient^∗^	Score
Age>60 years	No	1.00	Reference	—	—	0
	Yes	2.20	1.20–4.02	0.010	0.79	1

Fever>37.3°C	No	1.00	Reference	—	—	0
	Yes	2.28	1.26–4.12	0.006	0.82	1.5

Guarding	No	1.00	Reference	—	—	0
	Yes	3.78	2.16–6.62	<0.001	1.33	2

PMN>75%	No	1.00	Reference	—	—	0
	Yes	2.31	1.33–8.52	0.046	0.83	1.5

Duration of pain to ER						
<12 hours		1.00	Reference	—	—	0
12–23 hours		0.58	0.10–3.37	0.542	0.54	1
>24 hours		6.60	2.28–19.16	0.001	1.89	3

*Note*. ^*∗*^ Coefficients from multivariable binary logistic regression. aOR, adjusted odds ratio; PMN, polymorphonuclear white blood cell.

**Table 3:** Probability categories in the RAMA WeRA Risk Score.

Probability categories	Score	Ruptured (+) (*n* = 77)		Nonruptured (−) (*n* = 403)		Sens	Spec	PPV	NPV	LHR+	95% CI	*p* value
		n	%	n	%							
Low	<2	0	0	45	11.17	—	88.8	—	82.3	0	—	<0.001
Moderate	2–6	31	40.26	296	73.45	40.3	26.6	9.5	70	0.55	0.41–0.72	<0.001
High	>6	46	59.74	62	15.38	59.7	84.6	38.9	91.7	3.88	2.90–5.21	<0.001

**Table 4:** Comparison of risk factors for ruptured appendicitis in suspected acute appendicitis patients by various studies.

Risk predictor	Sirikurnpiboon and A mornpornchareon[5]	Sheu et al. [6]	RAMA WeRA study
Age > 60 years	—	1.05 (1.02–1.07)	2.2 (1.20–4.02)
Fever	1.97 (1.03–3.78)	2.59 (1.78–3.77)	2.28 (1.26–4.12)
Guarding	—	—	3.78 (2.16–6.62)
PMN	—	2.34 (1.27–4.32)	2.31 (1.33–8.52)
Duration to ER	4.21 (2.22–7.98)	1.23 (1.11–1.36)	6.60 (2.28–19.16)
Anorexia	1.90 (1.03–3.52)	2.03 (1.38–2.99)	1.10 (0.62–1.98)
Gender, male	2.47 (1.31–4.63)	1.96 (1.02–1.07)	1.08 (0.61–1.93)

## Data Availability

The data used to support the findings of this study are available from the corresponding author upon request.
